# Magnesium in Combinatorial With Valproic Acid Suppressed the Proliferation and Migration of Human Bladder Cancer Cells 

**DOI:** 10.3389/fonc.2020.589112

**Published:** 2020-12-11

**Authors:** Tianye Li, Yang Yu, Hang Shi, Yuhua Cao, Xiangfu Liu, Zhenzhen Hao, Yuping Ren, Gaowu Qin, Yongye Huang, Bing Wang

**Affiliations:** ^1^College of Life and Health Sciences, Northeastern University, Shenyang, China; ^2^Key Laboratory for Anisotropy and Texture of Materials (Ministry of Education), School of Materials Science and Engineering, Northeastern University, Shenyang, China

**Keywords:** magnesium, apoptosis, autophagy, valproic acid, combinatorial treatment

## Abstract

Magnesium, the second most predominant intracellular cation, plays a crucial role in many physiological functions; magnesium-based biomaterials have been widely used in clinical application. In a variety of cancer types, the high intracellular concentration of magnesium contributes to cancer initiation and progression. Therefore, we initiated this study to investigate the likelihood of confounding magnesium with cancer therapy. In this study, the anti-tumor activity of magnesium and underlying mechanisms were assessed in bladder cancer both *in vitro* and *in vivo*. The results indicated that the proliferation of bladder cancer cells was inhibited by treatment with a high concentration of MgCl_2_ or MgSO_4_. The apoptosis, G0/G1 cell cycle arrest, autophagy, and ER stress were promoted following treatment with MgCl_2_. However, the migratory ability of MgCl_2_ treated cells was similar to that of control cells, as revealed by the trans-well assay. Besides, no significant difference was observed in the proportion of CD44 or CD133 positive cells between the control and MgCl_2_ treated cells. Thus, to improve the therapeutic effect of magnesium, VPA was used to treat cancer cells in combination with MgCl_2_. As expected, combination treatment with MgCl_2_ and VPA could markedly reduce proliferation, migration, and *in vivo* tumorigenicity of UC3 cells. Moreover, the Wnt signaling was down-regulated, and ERK signaling was activated in the cells treated with combination treatment. In conclusion, the accurate utilization of MgCl_2_ in targeting autophagy might be beneficial in cancer therapy. Although further studies are warranted, the combination treatment of MgCl_2_ with VPA is an effective strategy to improve the outcome of chemotherapy.

## Introduction

Bladder cancer ranks the second most frequent urological malignancy worldwide, with an estimated 549,393 newly diagnosed and approximately 199,922 deaths each year (1). Owing to high recurrence rates, periodic cystoscopic tumor surveillance strategies, and high treatment expenses, the lifetime management of bladder cancer remains very expensive (2). Chemotherapy continues to be the standard of care in patients with unresectable and metastatic bladder cancer (3, 4). Notably, cisplatin-based chemotherapy, a standard first-line treatment of choice based on studies demonstrating improved survival outcomes, has been the mainstay for bladder cancer for decades (5, 6). However, patients who receive traditional chemotherapeutic regimens might suffer from the significant risk of off-target toxicity and the development of resistance to drugs. To overcome these limitations, in recent years, many alternative strategies, including nanotechnology, have been developed to improve the therapeutic outcome of chemotherapy (7). Nanotherapy with the favorable characteristics of improved circulation and reduced toxicity has been implicated as one of the most promising strategies in clinical settings. Of note, the application of a yolk-shell Fe_3_O_4_@MgSiO_3_ nanoplatform with the polymerpoly (ethylene glycol) and folic acid modifications has been shown to effectively circumvent multidrug resistance in cancer cells (8). In bladder cancer, a combination of Mg^2+^-Catechin nanocomposite particles and siRNA has been suggested to be a promising therapeutic modality of combining chemotherapy with gene therapy to acquire higher therapeutic efficacy (9). Recently, nanocomposite particles have received significant attention as useful drug carriers for cancer therapy. However, whether the component of the nanocomposite particle itself plays a role in cancer therapy remains elusive. Magnesium ions (Mg^2+^), one of the most common components used in the fabrication of nanocomposites. Therefore, in this study, we made an attempt to determine the anti-tumor effect of magnesium in bladder cancer.

Magnesium (Mg^2+^) is an essential mineral micronutrient that functions as a cofactor for the activation of a variety of transporters and enzymes, and required for the physiologic functions of various organs. Green vegetables, including beans, broccoli, have a high content of magnesium; besides, almonds, bananas, cashews, egg yolk, flaxseed, including other nuts, oatmeal, pumpkin seeds, sesame seeds, soybeans, and whole grains are rich in magnesium (10). Intracellular Mg^2+^ may also act as a secondary messenger and regulate the activities of a vast array of enzymes and participate in the metabolism of various nutrients (11, 12). In humans, the total serum magnesium ranges 0.70 to 1.10 mmol/L (13). Magnesium represents the second most abundant intracellular cation and the fourth most common cation in the human body. Furthermore, magnesium is predominantly involved in many physiologic pathways, energy production, and it also exhibits structural functions. Moreover, magnesium and its alloys have been identified as promising implant materials; besides, the treatment with magnesium implants benefited almost all patients, including patients with cancer (14). In 1904, Payr suggested the use of magnesium for the treatment of cavernous haemangioma and large-vessel aneurysms therapy (14). Subsequent studies also indicated that magnesium alloys exhibit an inhibitory effect on tumor growth (15–17). Accumulative pieces of evidence have indicated the complex relationship between magnesium and cancer. Both epidemiological and clinical studies suggested that magnesium deficiency is associated with an increased risk of tumorigenesis (18, 19). In breast cancer, the cancer cells promote the expression of magnesium transport channels to enhance the intracellular concentration of the mineral, thereby meeting the increasing energy demand of cancer cells (20). Furthermore, reduced serum levels of Mg^2+^ is frequently associated in patients with solid tumors and is frequently correlated with the advanced malignancy. Moreover, several cycles of chemotherapy may also lead to reduced serum levels of Mg^2+^ in patients with cancer. In addition, in magnesium-deficient mice, tumor growth was inhibited at its primary site; however, it was increased in metastatic colonization (10). All these pieces of evidence indicated that the role of magnesium in tumorigenesis remains elusive and complex. Thus, it becomes imperative better to understand the vital function of magnesium in tumor development. Therefore, the present study investigated the anti-tumor activity of magnesium and assessed the underlying mechanisms in bladder cancer both *in vitro* and *in vivo*.

It appears that magnesium plays a protective role at the early stage of carcinogenesis, but contributes to the proliferation of existing tumors at the later stages (19). Combination therapy has been considered as the standard first-line treatment to improve the clinical outcome in several malignancies. Combination therapy with anticancer drugs has been shown to be effective in inducing the synergistic drug actions, prolonging the onset of chemoresistance, and enhancing the immunogenicity of cancer cells (21, 22). Histone deacetylase (HDAC) inhibitors represent a group of potent epigenetic modulators that are known to exhibit significant potential effects in cancer treatment. Valproic acid (VPA), an HDAC inhibitor, has emerged as a potent drug for cancer therapy in recent years and has shown promising antitumor effects in a variety of *in vitro* and *in vivo* systems. In early clinical trials, VPA alone or in combination with other agents has revealed encouraging results (23). Furthermore, many *in vitro* and *in vivo* studies suggest that VPA exhibits the characteristic of high effectiveness and relatively low toxicity profile. Considering these benefits of VPA, the present study also examined the synergistic effect of the combination of VPA and magnesium in cancer therapy.

Considering these evidences, we initiated this study to investigate the possibility of confounding magnesium with cancer therapy. In this study, the anti-tumor activity of magnesium and/or in combination with VPA and underlying mechanisms were assessed in bladder cancer both *in vitro* and *in vivo*.

## Materials and Methods

### Cell Cultures and Chemicals

Human bladder cancer cell lines (UM-UC3 and UM-UC5), immortalized normal gastric cell line (Ges1), gastric cancer cell line (MKN1), and HEK-293 cells were cultured in Dulbecco’s modified Eagle’s medium (DMEM) supplemented with 10% fetal bovine serum (FBS), 1% nonessential amino acid, 1% Glutamine, 100 U/ml streptomycin, and 100 U/ml penicillin in a humidified atmosphere of 5% CO_2_ at 37°C. Unless otherwise specified, all the chemicals were purchased from Sigma-Aldrich (St. Louis, MO, USA). For ERK inhibition, cells were pre-treated with U0126 (Selleck Chemicals, Houston, TX, USA) for 2 h followed by treatment with MgCl_2_ for 24 h. For IRE1α inhibition, cells were treated with STF083010 (Selleck Chemicals, Houston, TX, USA) for 24 h.

### Cell Viability Assay

In brief, cancer cells were seeded into 6-well plates at a density of 1.2×10^5^ cells/well and then treated with different concentrations of magnesium for 24 h. Subsequently, cells were rinsed with PBS and digested with trypsin containing EDTA. Cell suspensions were centrifuged by at 100 g for 5 min and then resuspended in 1 ml PBS. Cell number in 100 μl PBS was counted using a BD Accuri C6 flow cytometer (BD Biosciences, San Diego, CA, USA). Finally, the IC_50_ was calculated as the concentration of MgCl_2_ that inhibited cell growth by 50%.

Viability of cells with MgCl_2_ or VPA alone and in combination was assessed with Cell Counting Kit-8 (CCK8; Bimake, Houston, TX, USA) assay. Briefly, cells were seeded into 96-well plates at a density of 2,000 cells/well and incubated in a humidified incubator of 5% CO_2_ at 37 °C. Then, a 200 μl culture medium containing MgCl_2_ and/or VPA was added to each well of the treatment group for 24 h. Following different treatments, 10 μl CCK-8 solution was added to each well, and cells were incubated for 2 h at 37°C. The cell viability was revealed by the absorbance (OD), which was measured at 450 nm using a microplate reader.

### Flow Cytometric Analyses

For cell cycle distribution analysis, cells were seeded at a density of 3×10^5^ cells/well in a 6 cm dish and cultured for 12 h to adhere to cells. Then, the cells were treated with MgCl_2_ and/or VPA for 24 h. They were then harvested by centrifugation at 100 g for 5 min, washed twice with PBS, fixed with ice-cold 70% ethanol (in PBS) at -20°C overnight. The fixed cells were stained in propidium iodide (1 mg/ml) and ribonuclease-A (10 g/ml) (PI/RNase; BD Biosciences) for 30 min at room temperature in the dark. Cell cycle distribution was assessed by flow cytometry (BD LSRFortessa, BD Biosciences). Data were analyzed by ModFit software, and the distribution of cells in different phases of the cell cycle was determined as a ratio of the fluorescent area of the appropriate peaks to the total fluorescent area.

Apoptosis was determined using Annexin V/PI assay. Cells were seeded in a 6 cm dish (3×10^5^ cells/well). After 12 h, the cells were treated with MgCl_2_ and/or VPA for 24 h. Cells were digested with trypsin and harvested by centrifugation at 100 g for 5 min. The cells were resuspended with 100 μl of 1× binding buffer containing 5 μl of Annexin V-FITC/PI for 15 min in the dark at room temperature. The fluorescence of the cells was quantified by flow cytometry (BD Biosciences, Franklin Lakes, NJ, USA) using a FITC signal detector and a PI signal detector.

For the detection of cancer stem cells, the expression profiles of CD133 and CD44 in cultured cells were analyzed by flow cytometry. Briefly, cancer cells were treated with or without MgCl_2_ for 24 h and cells were then digested by trypsin. Following incubation, the suspension was centrifuged, and cells were suspended in PBS. The cell suspension was incubated with FITC-conjugated anti-CD44 or PE-conjugated anti-CD133 at 4°C for 30 min in the dark. Labeled cells were resuspended in PBS and analyzed by flow cytometer (BD Biotechnology).

### RNA Extraction and Quantitative Real-Time Polymerase Chain Reaction (qRT-PCR)

The total RNA from cancer cells was extracted with TRIzol reagent (Tiangen Biotech, Beijing, China) according to the manufacture’s protocol. One μg of total RNA was reverse transcribed into complementary DNA (cDNA) using All-in-One cDNA synthesis SuperMix (Bimake, Houston, TX, USA) kit according to the manufacturer’s instructions. Quantitative real-time PCR (qRT-PCR) was performed with 2× SYBR Green qPCR Master Mix (Bimake, Houston, TX, USA) following the manufacturer’s protocol on a CFX96 real-time PCR detection system. The amplification program was as follows: initial denaturation at 95°C for 15 min, followed by 45 cycles of denaturation at 95°C for 10 s, annealing at 60°C for 30 s and extension at 72°C for 20 s; final extension at 72°C for 10 min. Primer sequences were listed in **Table S1**. Glyceraldehyde phosphatedehydrogenase (GAPDH) or β-actin was used as an internal control for the normalization of gene expression data. The relative expression was calculated using the 2−^ΔΔCt^ method.

### Western Blotting

Cells treated with MgCl_2_ and/or VPA were collected and lysed with RIPA lysis buffer supplemented with protease inhibitor cocktail. Cell suspensions were then centrifuged to collect clear lysates in the supernatant and stored at -20°C. The protein contents were quantitated using the bicinchoninic acid (BCA) protein assay kit (Beyotime, Shanghai, China). Equal amounts (20 μg) of cell extracts were resolved by SDS-PAGE, and transferred to a PVDF membrane, blocked with a 5% non-fat milk solution for 1 h at room temperature, and incubated with primary monoclonal antibodies (as listed in **Table S2**) overnight at 4°C. The membranes were then incubated with the appropriate HRP-conjugated secondary antibodies at room temperature for 1 h; the immunoblots were visualized with an enhanced chemiluminescence detection system (Millipore, Boston, USA).

### Wound Healing and Migration Assay

The migration of the cells was assessed using a wound-healing assay. Briefly, cells with or without MgCl_2_ treatment were cultured in 6-well plates and cultured to form a tight monolayer. The monolayers were scratched with a 200 μl sterile pipette tip and washed with PBS. Subsequently, the cells were then cultured in serum-free media. After 0, 24, and 48 h, cellular migration toward the scratched area was photographed using an inverted microscope.

For the migration assay, a 24-well Transwell (8-μm pore size) was used. Briefly, 2× 10^4^ cells suspended in 200 μl serum-free DMEM were seeded to the upper chamber of each well, 600 μl of FBS-containing medium was added to the bottom chamber. After incubation for 24 h, cells that migrated to the lower membrane of the chamber were fixed with methanol for 30 min at room temperature and stained with 0.2% crystal violet for 20 min. Cells were then counted in five randomly selected fields (at ×200 magnification) under an inverted microscope, and the average cell number per view was calculated. All experiments were performed in triplicate.

### Colony Formation Assay

Following treatment with MgCl_2_, cells following treatment MgCl_2_ with were seeded into six-well plates at 1,000 cells/well and cultured for 20 days in a humidified atmosphere of 5% CO_2_ at 37°C to allow for colony growth. At the assay endpoint, the cells were washed gently with PBS and then fixed with paraformaldehyde for 30 min and stained with 0.1% crystal violet solution for 15 min. Stained colonies with a diameter larger than 50 μm were counted and photographed under microscope.

### Subcutaneous Tumorigenesis in Nude Mice

The animals were cared for in accordance with the Guide for the care and use of laboratory animals in China. All experimental procedures were approved by the Animal Care and Use Committee of the Northeastern University Committee, China.

Four-week-old male BALB/c nude mice were procured from Charles River (Beijing, China). The mice were grouped-housed under specific pathogen-free conditions, at a temperature of 24°C with a relative humidity of 50–60%, under a 12-h-light/12-h-dark schedule. Animals were provided ad libitum access to standard food and drinking water. All the mice were healthy and had no infection during the experimental period. All surgical procedures were performed under aseptic conditions. UC3 cells (2 × 10^7^ cells/ml) were injected into the right super lateral subcutaneous tissue of the nude mice. Tumor growth was measured with calipers every 4 days, and tumor volume was calculated according to the following equation: tumor volume (mm^3^) = 0.5 × the longest diameter × the shortest diameter^2^. When the mean tumor volume reached approximately 100 mm^3^, mice were randomized into the vehicle control group (received 100 μl physiological saline solution/2 days), MgCl_2_ treated group (50 mg/kg/2 days), VPA treated group (100 mg/kg/2 days), and combination treatment group (50 mg MgCl_2_/kg/2 days+ 100 mg VPA/kg/2 days). The drug was administered by intraperitoneal injection. At the termination of the experiment, the mice were euthanized by cervical dislocation, and tumors were harvested and weighed.

### Statistical Analysis

Data were expressed as means ± Standard Error of the Mean (SEM). The differences between the two groups were assessed using the unpaired Student’s *t*-test. Analysis of variance (ANOVA) was performed for multiple comparisons. Statistical analyses were performed using the SPSS 16.0 software (SPSS Inc., Chicago, IL, USA). A p-value of <0.05 was considered to be statistically significant. Each experiment was conducted in triplicate and repeated three times.

## Results

### Magnesium Inhibited the Growth of Bladder Cancer Cells UC3 and UC5

**Figure 1:**
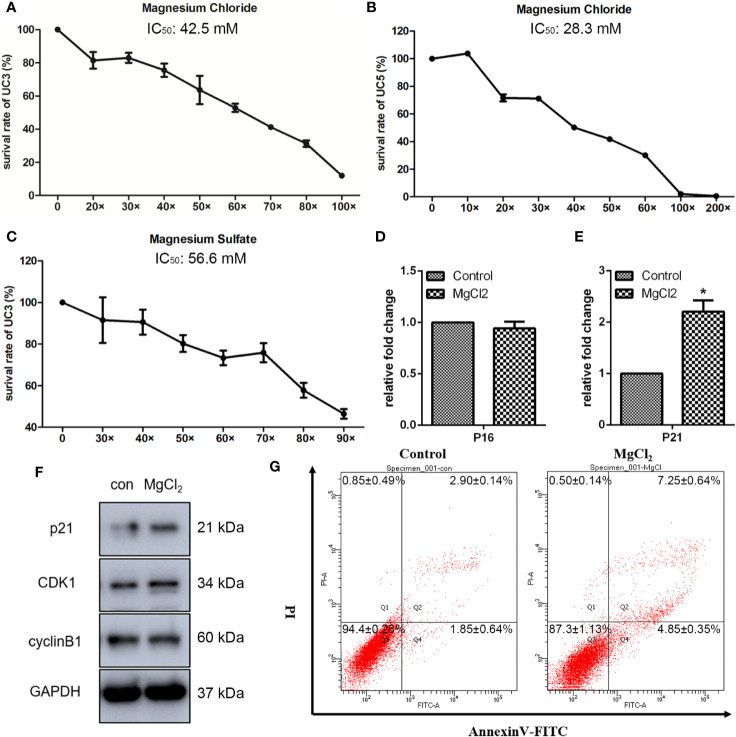
Magnesium inhibited the proliferation of bladder cancer cells. **(A)** The survival rate of UC3 cells at different concentrations of MgCl_2_ for 24 h. **(B)** The survival rate of UC5 cells at different concentrations of MgCl_2_ for 24 h. **(C)** The survival rate of UC3 cells at different concentrations of MgSO_4_ for 24 h. **(D, E)** mRNA expression of P16 and P21 as determined by qRT-PCR. Data were expressed as mean ± SEM of duplicate experiments. The differences between the two groups were assessed using the unpaired Student’s t-test. *p < 0.05 versus control. **(F)** Protein expression as revealed by Western blot analysis. **(G)** Apoptosis was evaluated by flow cytometry using double staining with Annexin V (Annexin V, horizontal line) and propidium iodide (PI, vertical line).

To determine the effects of magnesium on the bladder cancer cell viability *in vitro*, we analyzed the impact of different concentrations of magnesium on the bladder cancer cell lines. It has been reported that a human normal serum reference range for magnesium level is 0.70 to 1.10 mmol/L (13). Thus, we used 0.70 mmol/L magnesium as the lower concentration, defined as 1×. As illustrated in **Figure 1A**, the survival rate of UC3 cells was markedly decreased by MgCl_2_ in a dose-dependent manner. The IC_50_ of MgCl_2_ in UC3 cells was approximately 42.5 mM. The inhibitory effect on the viability of UC5 was also assessed. Results showed that the IC_50_ of MgCl_2_ in UC5 cells was about 28.3 mM (**Figure 1B**), indicating that UC5 cells were more sensitive to treatment with MgCl_2_. To further investigate the functions of magnesium in bladder cancer cells, the survival rate of UC3 cells with MgSO_4_ exposure was evaluated. The IC_50_ of MgSO_4_ in UC3 cells was about 56.6 mM (**Figure 1C**), possibly suggesting that magnesium played an essential role in tumor growth. To better illustrate the cytotoxicity of magnesium, a nontumorigenic HEK-293 (kidney) cell line was applied. The IC_50_ of MgCl_2_ in HEK-293 cells was about 60.22 mM which was higher than that in UC3 and UC5 cells (**Figure S1**). Based on these results, the subsequent experiments were performed using 42.5 mM MgCl_2_ in UC3 cells.

### Effect of MgCl_2_ on Canonical Cell Death Modes

Inhibition of cell proliferation is often associated with cell cycle arrest during chemotherapy. Many genes related to cyclin were investigated. To further characterize the impact of MgCl_2_ treatment, detailed analyses of cell-cycle distribution were performed. As anticipated, both qRT-PCR and Western blot assay results revealed that p21 expression was markedly increased following treatment with MgCl_2_ (**Figures 1D–F** and **S2A**). Surprisingly, there was no significant differences in the expression of p16, CDK1, and cyclin B1 between the control and MgCl_2_ treated cells, indicating that the cell cycle distribution remains mostly unaffected by treatment with MgCl_2_.

**Figure 2:**
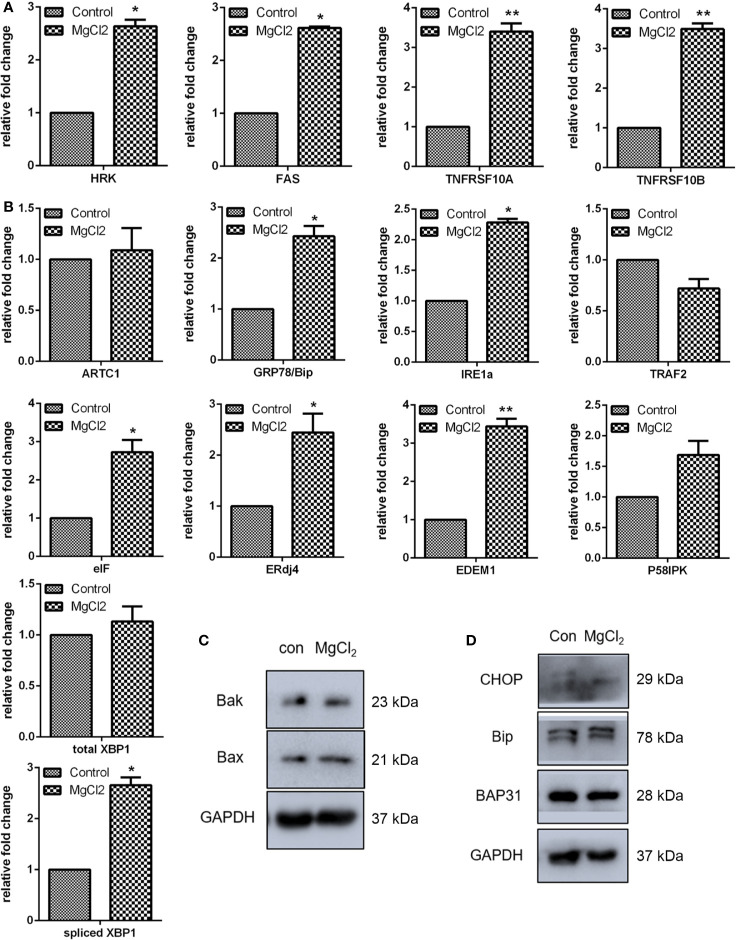
MgCl_2_ affected apoptosis and ER stress in bladder cancer cells UC3. **(A)** Expression of apoptosis-related genes as determined by qRT-PCR. **(B)** ER stress-related gene expression as revealed via qRT-PCR. **(C)** Protein expression of apoptosis-related genes as revealed via western analysis. **(D)** Protein expression of ER stress-related genes as determined by Western blot analysis. Data were expressed as mean ± SEM of duplicate experiments. The differences between the two groups were assessed using the unpaired Student’s t-test. *p < 0.05 versus control, and **p < 0.01 versus control.

To further explore the roles of MgCl_2_ in bladder cancer, detailed analyses of canonical cell death modes, including apoptosis, necrosis, and autophagic cell death were performed. Flow cytometry was performed to analyze the apoptosis of cells treated with MgCl_2_. The results demonstrated that the apoptotic rate in the control cells was 4.2% and in the MgCl_2_ treated cells were augmented to 12.8% (**Figure 1G**). The results of qRT-PCR assay revealed that the expression of HRK, FAS, TNFRSF10A, and TNFRSF10B was up-regulated in MgCl_2_ treated cells (**Figure 2A**). Western blot assay also further confirmed the increased expression of apoptosis-related genes. The expression of Bak and Bax was slightly enhanced in the cells treated with MgCl_2_ (**Figure 2C** and **S2B**). Western blot analysis of cytosolic *vs.* mitochondrial extracts revealed that Bax and Bak accumulated in mitochondria (**Figure S3**), further confirming that apoptosis was triggered by a high concentration of MgCl_2_.

Furthermore, a close relationship exists between apoptotic cell death and ER stress. To confirm our hypothesis that MgCl_2_ induces apoptosis through ER stress, the expression of ER stress-related genes (ATRC1, Bip, IRE1α, TRAF2, eIF, ERdj4, EDEM1, and P58IPK) was analyzed. As revealed by qRT-PCR, increased expression of Bip, IRE1α, eIF, ERdj4, and EDEM1 were found in MgCl_2_ treated cells (**Figure 2B**). To further confirm whether MgCl_2_ can induce ER stress, a Western blot assay was performed to detect the expression of CHOP, Bip, and BAP31. As presented in **Figure 2D**, treatment with MgCl_2_ enhanced the expression of CHOP remarkably. These results indicated that MgCl_2_ could induce ER stress, which might play a crucial role in MgCl_2_-induced apoptosis of UC3 cells.

**Figure 3:**
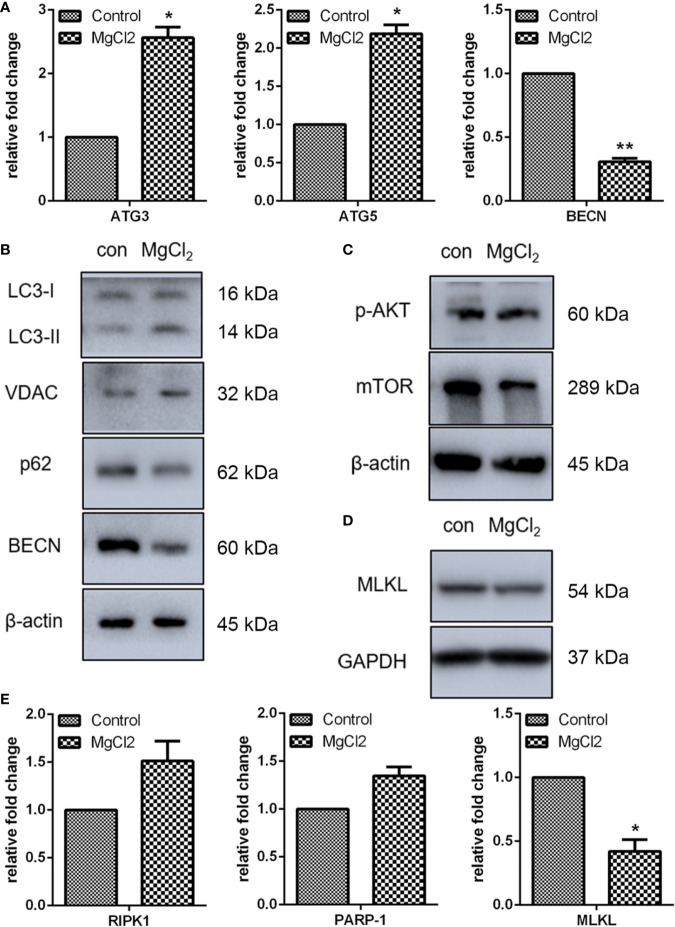
MgCl_2_ affected autophagy and necroptosis in UC3 cells. **(A)** mRNA expression of autophagy-associated genes as revealed by qRT-PCR. **(B)** Protein expression of autophagy marker genes as determined by Western blot analysis. **(C)** Evaluation of Akt phosphorylation (p-Akt) and mTOR as determined by Western blot analysis. **(D)** Expression of MLKL as determined by Western blot analysis. **(E)** Gene expression related to necroptosis, as revealed by qRT-PCR. The differences between the two groups were assessed using the unpaired Student’s t-test. Data were expressed as mean ± SEM of duplicate experiments. *p < 0.05 versus control, and **p < 0.01 versus control.

Autophagy plays a protective role in the resistance to apoptosis under various apoptotic conditions (24). Autophagy can function as an alternative cell death mechanism defined as programmed cell death type II. Besides, during the progression of autophagy, the cleavage of LC3 is increased. As determined by Western blot assay, the expression of LC3-II was up-regulated in MgCl_2_ treated cells (**Figure 3B** and **S2C**). However, the expression of other autophagy marker p62/SQSTM was down-regulated following treatment with MgCl_2_. These results indicated that autophagy was induced by MgCl_2_ therapy. Furthermore, additional autophagy-associated genes were also analyzed. Results showed that the expression of ATG3, ATG5 and VDAC were increased following treatment with MgCl_2_ (**Figures 3A, B**), further confirming the role of autophagy in MgCl_2_ therapy. However, both qRT-PCR and Western blot assay revealed that the BECN was decreased in the cells treated with MgCl_2_, indicating that MgCl_2_ mediated autophagy was independent of BECN expression. Akt-mTOR pathway is essential for the induction of autophagy, and the results of Western blot analysis revealed that both p-Akt and mTOR was attenuated following treatment with MgCl_2_ (**Figure 3C**). Taken together, the results implicated that MgCl_2_ triggered BECN independent autophagy via Akt-mTOR signaling.

The present study also analyzed the expression of necroptosis related genes RIPK1, PARP1, and MLKL. Results of qRT-PCR indicated that there were no significant differences in the expression of RIPK1 and PARP1 between the control and MgCl_2_ treated cells (**Figure 3E**). However, MLKL mRNA level was decreased in the cells treated with MgCl_2_. Western blot assay further confirmed the decreased expression of MLKL in the MgCl_2_ treated cells (**Figure 3D**). Collectively, the results indicated that necroptosis was inhibited with MgCl_2_ treatment in bladder cancer cells.

### Effect of MgCl_2_ on Wnt and Ras/Raf/MEK/ERK Signaling

**Figure 4:**
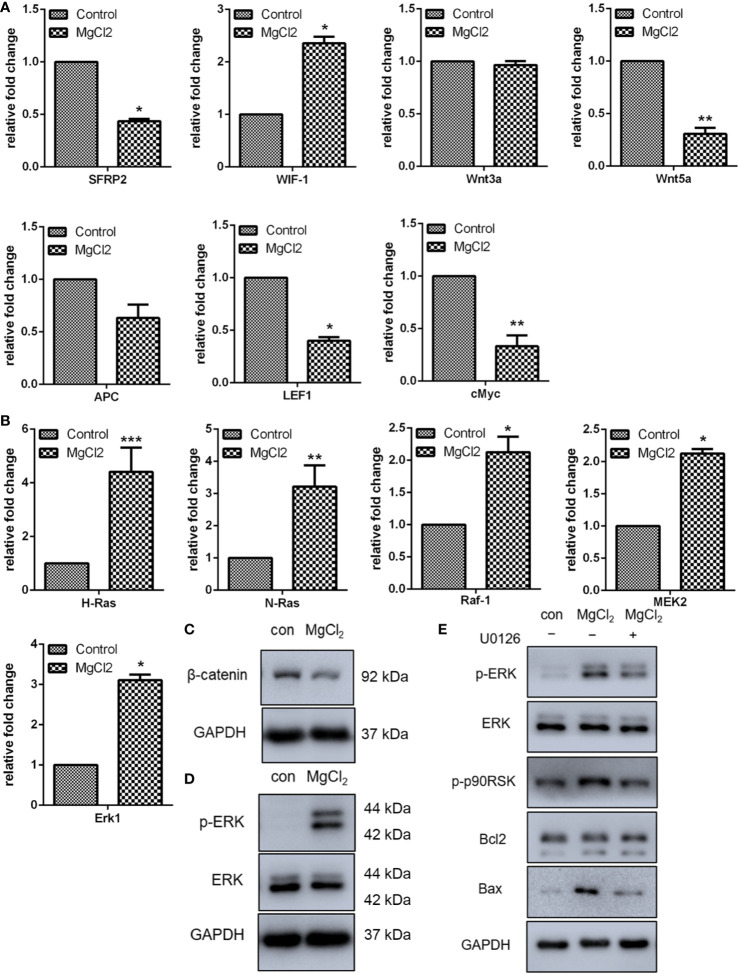
MgCl_2_ regulated Wnt and ERK signaling pathways in UC3 cells. **(A)** Wnt signaling genes expression analysis by qRT-PCR. **(B)** mRNA expression of ERK signaling was determined by qRT-PCR. **(C)** Protein expression of β-catenin as determined by Western blot analysis. **(D)** Protein expression of ERK and ERK phosphorylation (p-ERK) as determined by Western blot analysis. **(E)** Protein expression as determined by Western blot analysis in cells treated with U0126. Data were expressed as mean ± SEM of duplicate experiments. The differences between the two groups were assessed using the unpaired Student’s t-test. *p < 0.05 versus control, **p < 0.01 versus control, and ***p < 0.001 versus control.

The Wnt signaling pathway has been implicated in tumorigenesis and tumor recurrence, and clinical trial of drugs targeting the Wnt pathway has shown promising results. Therefore, in the present study, we analyzed the expression of Wnt signaling in bladder cancer cells *in vitro*. Using qRT-PCR, we analyzed the expression of SFRP2, WIF-1, Wnt3a, Wnt5a, APC, LEF1, and c-Myc. The results indicated that the expression of SFRP2, Wnt5a, LEF1, and c-Myc was down-regulated following treatment with MgCl_2_ (**Figure 4A**). While the treatment with MgCl_2_ up-regulated the expression of WIF-1. However, there was no significant difference in the expression of Wnt3a and APC between the control and MgCl_2_ treated group. Possibly, the Wnt signaling pathway was inhibited by MgCl_2_. To support this hypothesis, we performed a Western blot assay to evaluate the expression of β-catenin, which is a pivotal component of the Wnt/β-catenin signaling pathway. The results indicated that the expression of β-catenin was down-regulated following treatment with MgCl_2_ (**Figure 4C**).

The Ras/Raf/MEK/ERK signaling is yet another important signaling pathway involved in carcinogenesis and progression. Thus, the expression of H-Ras, N-Ras, Raf-1, and MEK2 was analyzed using qRT-PCR, and results showed that all these genes were activated following treatment with MgCl_2_ (**Figure 4B**). The expression of ERK was then examined using Western blot assay. As represented in **Figure 4D**, there was no significant difference in the expression of ERK between the control and MgCl_2_ treated group. However, ERK phosphorylation was enhanced in the MgCl_2_ treated cells. To further investigate the effect of MgCl_2_ on Ras/Raf/MEK/ERK signaling, the pretreatment with mitogen−activation protein kinase kinase inhibitor, U0126, was performed. As anticipated, pretreatment with U0126 suppressed the enhanced effect of MgCl_2_ on ERK phosphorylation (**Figure 4E**). Furthermore, the upregulation of Bax and LC3-II induced by MgCl_2_ treatment was also inhibited in cells pre-treated with U0126 (**Figure 4E** and **S3**), possibly indicating that MgCl_2_ induced apoptosis and autophagy could be prevented through the inhibition of Ras/Raf/MEK/ERK signaling.

### MgCl_2_ Exerted Minor Effects on the Migration and Stemness

**Figure 5:**
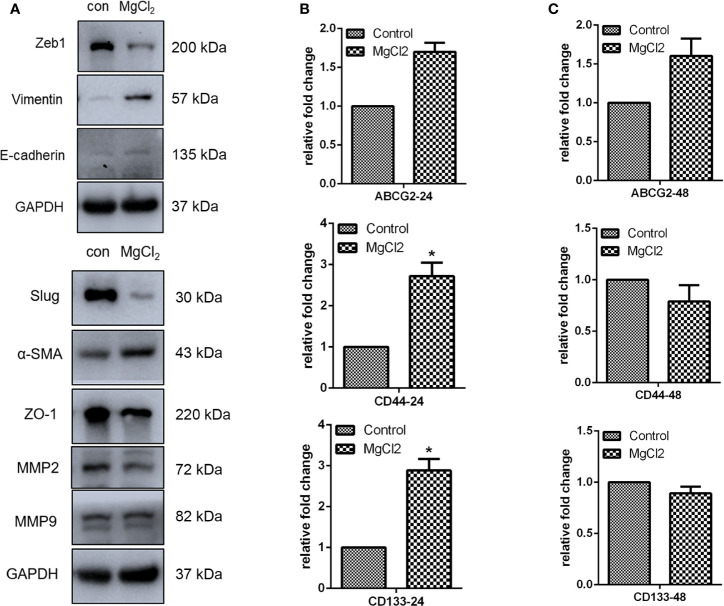
MgCl_2_ affected the migration of UC3 cells. **(A)** Protein expression of EMT related genes as evaluated by Western blot analysis. **(B)** mRNA expression of cancer stem cell marker genes was determined by qRT-PCR in the cells treated with MgCl_2_ for 24 h. **(C)** mRNA expression of cancer stem cell marker genes was determined by qRT-PCR in the cells treated with MgCl_2_ for 48 h. Data were expressed as mean ± SEM of duplicate experiments. The differences between the two groups were assessed using the unpaired Student’s t-test. *p < 0.05 versus control.

**Figure 6:**
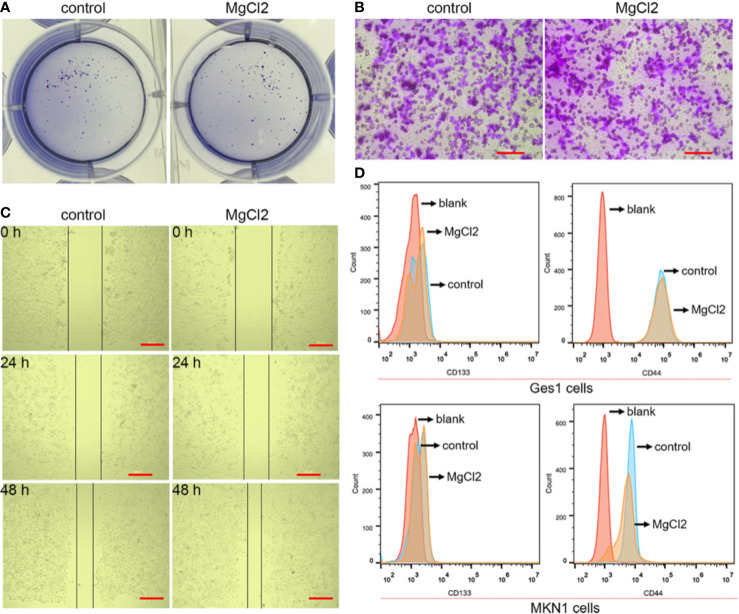
Effect of MgCl_2_ on the proliferation, migration, and stemness in the bladder cancer cells. **(A)** Colony formation in the UC3 cells treated with MgCl_2_. **(B)** Trans-well assay of the UC3 cells treated with MgCl_2_. Scale bars indicate 100 μm. **(C)** Results of the wound healing assay of the UC3 cells treated with MgCl_2_. Scale bars indicate 200 μm. **(D)** The proportion of CD44 and CD133 positive cells in MKN1 or Ges1 cells treated with MgCl_2_ by flow cytometry.

The epithelial-to-mesenchymal transition (EMT), together with its reverse process mesenchymal-epithelial transition (MET), plays an important role in cancer progression and metastasis. In this study, E-cadherin was found to be up-regulated in the MgCl_2_ treated cells (**Figure 5A**). However, the expression of epithelial gene ZO-1 was downregulated following treatment with MgCl_2_. Furthermore, we performed a Western blot assay to analyze the expression of mesenchymal genes. MgCl_2_ suppressed the expression of Zeb1 and Slug; however, it enhanced the expression of Vimentin and α-SMA. It was found that the EMT process was disrupted by treatment with MgCl_2_. MMPs have been known to degrade the extracellular matrix, thus promoting migration and invasion of cancer cells. The results of Western blot analysis revealed that MgCl_2_ reduced the expression of MMP2 and MMP9. However, treatment with MgCl_2_ was shown to exert no significant effects on colony formation, migration, and invasion (**Figures 6A–C**).

The EMT process is suggested to be closely associated with the acquisition of cancer stemness properties. Therefore, we examined the expression of genes related to cancer stemness. Surprisingly, the expression of CD44 and CD133 was promoted by treatment with MgCl_2_ (**Figure 5B**). Next, we analyzed whether prolonged treatment duration would have an impact on the expression profile of these genes. At 48 h post-treatment with MgCl_2_, there were no significant differences in the expression profiles of ABCG2, CD44, and CD133 between the control and MgCl_2_ treated cells (**Figure 5C**). To better illustrate the effect of MgCl_2_ on the cancer stemness, we performed flow cytometry to analyze the expression of cancer stem cells markers CD44 and CD133 in immortalized normal gastric cells Ges1 and gastric cancer cells MKN1. In both Ges1 and MKN1 cells, treatment with MgCl_2_ did not markedly alter the expression of CD44 and CD133 (**Figure 6D**). Considering the gene expression profile and cell biology experiments, it was apparent that the migration and cancer stemness was not significantly inhibited by treatment with MgCl_2_.

### VPA Enhanced the Anti-Tumor Effect of MgCl_2_

**Figure 7:**
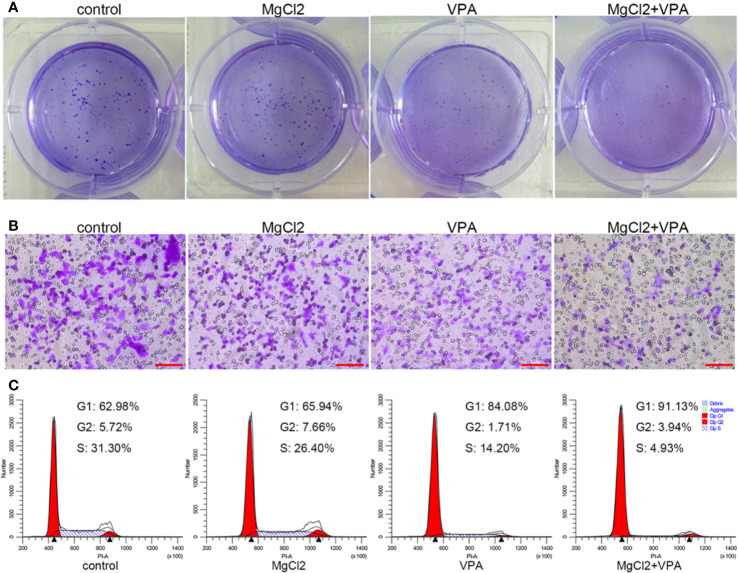
MgCl_2_, in combination with VPA, inhibited the proliferation, migration, and cell cycle distribution in UC3 cells. **(A)** Colony formation in the cells following the combination treatment. **(B)** Trans-well assay of the cells with combination treatment. Scale bars indicate 100 μm. **(C)** Cell cycle distribution was evaluated by flow cytometry based on PI staining.

Results indicated that MgCl_2_ treatment reduced the acH4K5 abundance in UC3 cells (**Figure S2E**). Many literatures have documented that enhancing acetylation could be useful in the prevention of cancer cell proliferation and metastasis. Therefore, to strengthen the anti-tumor function of MgCl_2_, cancer cells were subjected to combination treatment with MgCl_2_ and VPA (5 mM). As illustrated in **Figure 7A**, cells with combined treatment of MgCl_2_ and VPA developed considerably lower numbers of colonies compared to control cells. Furthermore, the number of cells with combined treatment that migrated through the trans-well membrane was also markedly lower than that of control cells (**Figure 7B**). These results confirmed that VPA could effectively contribute to reinforce the anti-tumor function of MgCl_2_.

**Figure 8:**
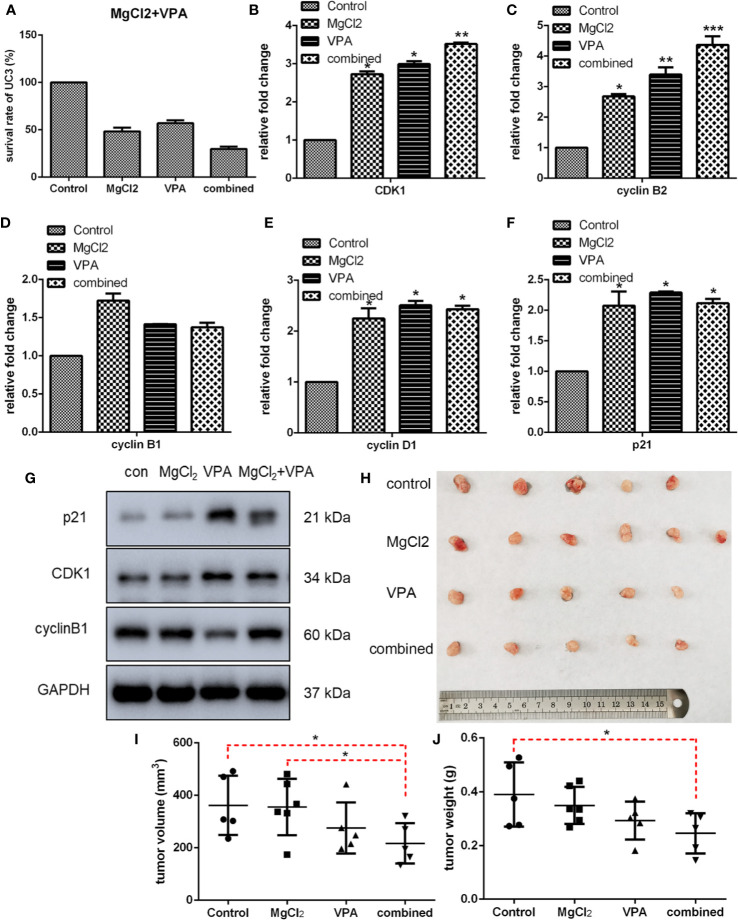
Combination treatment with MgCl_2_ and VPA affected cell survival, expression of genes related to cell cycle progression, and *in vivo* tumorigenicity of UC3 cells. **(A)** The survival rate of cells with combination treatment as determined by CCK-8 assay. **(B–F)** mRNA expression of genes related to cell cycle progression was investigated by qRT-PCR. Data were expressed as mean ± SEM of duplicate experiments. **(G)** Western blot analysis of genes related to cell cycle progression. **(H)** UC3 cells were injected into BALB/c nude mice, as indicated. After therapy with MgCl_2_ and/or VPA for 14 days, mice were sacrificed to harvest tumors. The tumor volume **(I)** and weight **(J)** were inhibited by combination treatment compared with the control group. The differences among multiple groups were assessed using the Analysis of variance (ANOVA). *p < 0.05 versus control, **p < 0.01 versus control, and ***p < 0.001 versus control.

Next, we performed CCK8 assay to determine the inhibitory effect of combined treatment on cell proliferation, and results indicated that a considerably lower survival rate was observed in the cells with combination treatment (**Figure 8A** and **S4A**). Furthermore, the suppressive effect of MgCl_2_ and/or VPA on *in vivo* tumorigenicity was also evaluated in UC3 cells tumor-bearing mice. The results revealed that MgCl_2_ and VPA in combination significantly reduced the tumor volume compared with control (216.95 ± 77.21 mm^3^ vs. 361.87 ± 113.21 mm^3^, *p* = 0.035; **Figures 8H, I**). Consistent with the above results, the tumor weight of UC3 cells tumor-bearing mice receiving combined treatment was also significantly lower than the control group (0.25 ± 0.07 g vs. 0.39 ± 0.12 g, *p* = 0.016; **Figures 8H, J**).

**Figure 9:**
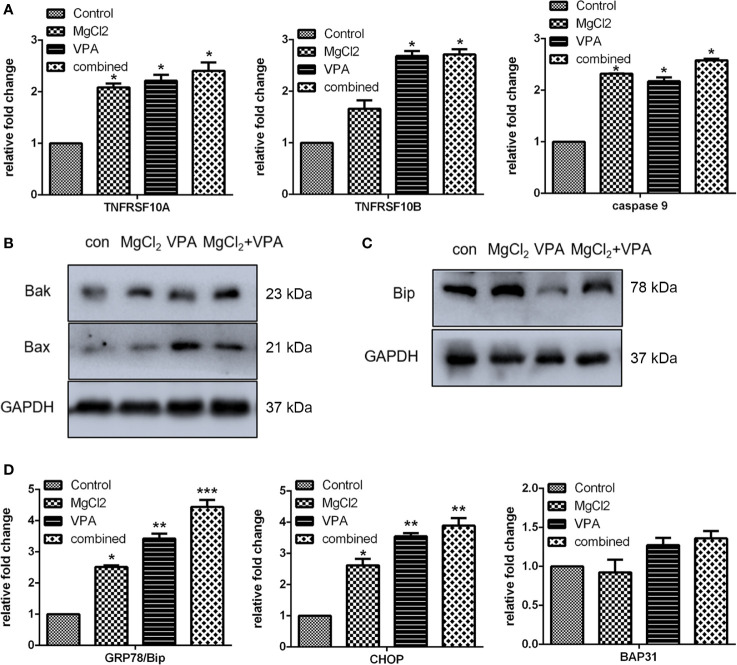
Expression of apoptosis or ER stress-associated genes in UC3 cells with combination treatment. **(A)** mRNA expression of apoptosis-related genes as determined by qRT-PCR. **(B)** Western blotting analysis for the expression of Bax and Bak. **(C)** Western blot analysis for the expression of Bip. **(D)** mRNA expression of genes related to ER stress was investigated by qRT-PCR. Data were expressed as mean ± SEM of duplicate experiments. The differences among multiple groups were assessed using the Analysis of variance (ANOVA). *p < 0.05 versus control, **p < 0.01 versus control, and ***p < 0.001 versus control.

Then, we further investigated the molecular mechanism underlying combination treatment was then investigated, and as revealed by flow cytometry, G0/G1 cell cycle arrest was most severe in the cells receiving combination treatment (**Figure 7C**). Consistent with the results of cell cycle analysis, cells with combination treatment exhibited increased expression of CDK1, cyclin B1, cyclin B2, cyclin D1, and p21 (**Figure 8** and **S4B**). Besides, the mRNA level of the apoptotic markers TNFRSF10A, TNFRSF10B, and caspase 9 were also increased in the cells receiving combination treatment (**Figure 9A**). Results of Western blot assay also indicated that the expression of Bak and Bax was also enhanced following combined treatment with MgCl_2_ and VPA (**Figure 9B** and **S4B**).

The expression of ER stress-related genes (Bip, CHOP, and BAP31) was also analyzed using qRT-PCR and Western blot assay. The mRNA expression of Bip and CHOP were up-regulated in cell treated with combination treatment (**Figure 9D** and **S4C**). The result of Western blot assays further confirmed that the Bip expression was markedly enhanced by combination treatment (**Figure 9C**). A specific IRE1α inhibitor STF083010 was applied to uncover the role of ER stress in combination treatment. As shown in **Figure S5**, the increasement in the expression of spliced XBP1 in the cells with combination treatment was inhibited by STF083010 as expected. Furthermore, the upregulation of TNFRSF10A in the cells with combination treatment was also prevented by STF083010, indicating that ER stress is possible a critical regulator of apoptosis induced by combination treatment.

**Figure 10:**
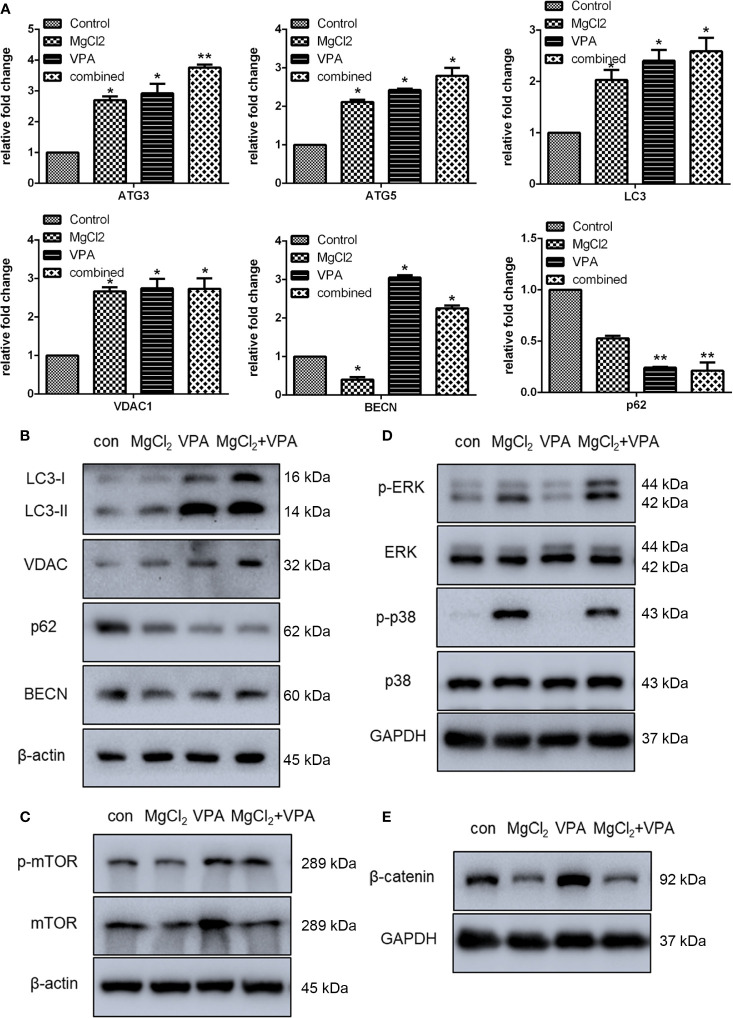
Combination treatment with MgCl_2_ and VPA regulated autophagy and many signaling pathways in the UC3 cells. **(A)** mRNA expression of autophagy-related genes as determined by qRT-PCR. **(B)** Western blot analysis for the expression of genes related to autophagy. **(C)** Protein expression of mTOR and phosphorylated mTOR (p-mTOR) as determined by Western blot analysis. **(D)** Protein expression of ERK signaling genes as determined by Western blot analysis. **(E)** Protein expression of β-catenin as determined by Western blot analysis. The differences among multiple groups were assessed using the Analysis of variance (ANOVA). *p < 0.05 versus control, and **p < 0.01 versus control.

To demonstrate whether VPA could enhance induced autophagy, the expression of ATG3, ATG5, LC3, VDAC, BECN, and p62 was examined. The expression of ATG3, ATG5, LC3, VDAC1, and BECN at the mRNA level was significantly enhanced with combination treatment (**Figure 10A**). Western blot analysis also revealed an increased level of LC3-II protein production in cells with combination treatment as compared to the control (**Figure 10B** and **S4D**). Results of Western blot analysis also showed that combination treatment promoted the VDAC expression. However, the expression of p62 was suppressed following treatment with MgCl_2_ and VPA, as indicated by qRT-PCR and Western blot analysis. In addition, the phosphorylation of mTOR was also up-regulated in the cells receiving combination treatment (**Figure 10C**), implying that induction of autophagy was reinforced by combination treatment.

Furthermore, we also found that the Ras/Raf/MEK/ERK signaling was activated, and the Wnt signaling was down-regulated following treatment with MgCl_2_. Therefore, we next investigated these signaling pathways in cells following combination treatment. The expression of ERK and p38MAPK were similar among the tested groups; however, the phosphorylation of ERK and p38MAPK were predominantly enhanced following combination treatment (**Figure 10D**). The expression of β-catenin was up-regulated in the cells with VPA treatment but was decreased in the cells with MgCl_2_ or combination treatment (**Figure 10E**). Collectively, these findings suggested that combination treatment inhibited cell proliferation, induced cell cycle arrest, enhanced apoptosis and autophagy, and suppressed migration through activation of ERK signaling and inhibition of the Wnt signaling pathway, thus VPA ameliorated the anti-tumor effect of MgCl_2_.

## Discussion

The present study demonstrated that treatment with MgCl_2_ could affect the proliferation and apoptosis of bladder cancer cells through the regulation of Ras/Raf/MEK/ERK and Wnt signaling pathways. However, the migratory ability and stemness of cancer cells were not markedly disrupted by treatment with MgCl_2_. Thus, when MgCl_2_ in combination with VPA was used, the migration of cancer cells was severely suppressed.

Accumulating pieces of evidence have reported that magnesium can act as a protective agent in colorectal carcinogenesis by suppressing c-Myc expression and ornithine decarboxylase function in the intestinal mucosa (25). In this study, we also found that the proliferation of bladder cancer cells was inhibited by different concentrations of magnesium. As indicated by flow cytometry analysis, treatment with MgCl_2_ could trigger apoptosis and induce cell cycle arrest. MgCl_2_ altered the expression of many genes associated with cell cycle and apoptosis. Notably, p21 was up-regulated following treatment with MgCl_2_. The p21 functions as a potent cyclin-dependent kinase inhibitor, up-regulation of which could induce cell cycle arrest (26). Besides, p21 can interact with various molecules, such as p53 and Myc, depending on different stimuli (26). In fact, due to interaction with different units, p21 could also function as an inhibitor of apoptosis, thus contributing to cancer development (26, 27). Therefore, combination therapy is expected to overcome challenges.

Autophagy, a lysosome-dependent cellular degradation program, is an evolutionary conserved catabolic process that provides cellular material to lysosomes for degradation to regulate the cellular homeostasis. A complex interplay between autophagy and apoptosis may contribute to cancer progression and therapeutic outcomes. A growing number of studies indicated that autophagy has a dichotomous role in the regulation of cancer (28, 29). In breast cancer, BECN and ATG7 mediated autophagy is considered as a means of evading apoptosis following DNA damage, thus prolonging cell survival (30). Thus, inhibition of autophagy to improve clinical outcomes in patients with cancer appears to be a favorable strategy. However, several studies have indicated that inhibition of autophagy may not confer benefit in cancer therapy as it may reduce anti-tumor T cell responses (28). In the present study, MgCl_2_ triggered autophagy through the regulation of Akt/mTOR signaling. Indeed, targeting autophagy represents a promising therapeutic strategy to circumvent resistance and improve the outcomes of chemotherapy (31). Therefore, the accurate utilization of MgCl_2_ in targeting autophagy might be beneficial in cancer therapy.

ER stress is a typical cellular stress response representing an evolutionarily conserved mechanism for maintaining cellular homeostasis. ER stress is closely associated with the induction of apoptosis and autophagy (32, 33). In this study, the splicing of XBP1 was induced with the MgCl_2_ treatment, indicating that the ER stress was triggered following treatment with MgCl_2_. Furthermore, treatment with MgCl_2_ up-regulated the expression of CHOP, Bip, IRE1α, eIF, ERdj4, EDEM1, and P58IPK. There are at least three pathways involving sensors of ER stress: (1) pancreatic endoplasmic reticulum kinase (PERK) phosphorylates eukaryotic initiation translation factor 2α (eIF2α); (2) translocation of activating transcription factor 6 (ATF6) from the ER to the Golgi and then the nucleus; and (3) inositol-requiring enzyme 1α (IRE1α) splices X-box-binding protein (XBP1) mRNA (34). Considering the significant upregulation of IRE1α and spliced XBP1 after MgCl_2_ treatment, we hypothesized that the IRE1α-XBP1 branch might involve in the treatment. Results of STF083010 treatment confirmed that blocking IRE1α could prevent the splicing of XBP1 and reduce the expression of TNFRSF10A. IRE1α can activate Jun-N-terminal kinase (JNK) to trigger autophagy or apoptosis (35). JNK mediated Bcl2 phosphorylation lead to the BECN/Bcl2 dissociation and autophagy induction in the early phase of ER stress, and sustained activation of JNK could trigger apoptosis due to prolonged ER stress (32). Conceivably, MgCl_2_ mediated the crosstalk between autophagy and apoptosis by modulating the expression of these genes.

Wnt signaling modulates cancer stem cells, metastasis, and immunity, is tightly associated with cancer (36). Inhibition of Wnt signaling is a universal strategy for cancer therapy, and some of Wnt signaling inhibitors are currently under clinical testing (36, 37). In this study, Wnt signaling was suppressed following treatment, possibly indicating that Wnt signaling was a critical target of the combination therapy. The Ras/RAF/MEK/ERK signaling pathway is another best-defined pathway in cancer biology (38). ERKs belong to the MAPK family of kinases, which also comprise of p38MAPK and JNK (39). ERKs are suggested as a double-edged sword in cancers which can activate pro-survival pathways contributing to cell proliferation and migration, or can function against cancer by regulating apoptosis, differentiation, and senescence (40). Increased ERK phosphorylation emerged in the cells with combination treatment in the present study. To uncover the role of Ras/RAF/MEK/ERK signaling pathway in magnesium treatment, the ERK inhibitor U0126 was applied. The Bax/Bcl2 ratio in MgCl_2_ treated cells was decreased by U0126, indicating that the Ras/RAF/MEK/ERK signaling pathway is critical for the apoptotic effect of MgCl_2_ treatment. In fact, U0126 has been found to block the apoptosis in many cancer cell types (41, 42). The down-regulation of Bax and cytochrome C in the mitochondrial protein fractions from cells with U0126 exposure further confirmed the role of Ras/RAF/MEK/ERK signaling pathway in MgCl_2_ induced apoptosis.

EMT enhances cell migration and metastasis of cancer cells (43, 44). As anticipated, E-cadherin expression was up-regulated following treatment with MgCl_2_. However, loss of E-cadherin conveys a poor prognosis in multiple human cancers, including bladder cancer (45). In most experimental models, E-cadherin is evaluated as an indicator of the occurrence of EMT (46). Considering these evidences, we suggested that treatment with MgCl_2_ could inhibit the EMT process. However, the expression of some of the marker genes was not apparent after treatment with MgCl_2_. Furthermore, the results of the present study also demonstrated that the migratory ability of cancer cells in control and MgCl_2_ treated group was similar. It is well recognized that migration is critical for cancer cell invasion and dissemination. Cancer stem cells, a subpopulation of cancers, harbor stemness that is perceived to considerably contribute to cancer metastasis, relapse, and therapy resistance (43). In previous studies, ABCG2, CD44, and CD133 were used to identify and isolate the bladder cancer stem cells (47, 48). In the present study, treatment with MgCl_2_ did not show an inhibitory effect on the expression of ABCG2, CD44, and CD133. In contrast, an up-regulation in the expression of CD44 and CD133 were presented in the cells treated with MgCl_2_ for 24 h. Possibly, MgCl_2_ did not have a favorable effect on the suppression of migration and stemness. Indeed, E-cadherin acts as a pleiotropic molecule that can contribute to either tumor-suppressing or tumor-promoting processes (49).

Combination therapy has been shown to improve clinical efficacy in many reports. VPA, a histone deacetylase inhibitor, has been widely used as an effective chemotherapeutic drug in a variety of cancers. VPA has been indicated to markedly promote the action of a number of chemotherapy agents in bladder cancer (50). In our previous study, the inhibitory effect on bladder cancer was found to be improved when melatonin was used in combination with VPA (51). In this study, combination treatment with MgCl_2_ and VPA revealed a potent ability in the inhibition of cell proliferation, migration, and *in vivo* tumorigenicity. The inhibitory effect of MgCl_2_ on proliferation and cell cycle was further strengthened by treatment with VPA. In fact, VPA has been shown to improve the anti-tumor effect of several first-line drugs, including Sorafenib and cisplatin (52, 53). In sum, further studies are warranted to improve the therapeutic effects of magnesium in cancer chemotherapy.

## Conclusion

Collectively, the findings of the present study indicated that combination treatment of MgCl_2_ with VPA inhibited cell proliferation, induced cell cycle arrest, enhanced apoptosis, and autophagy, and suppressed migration through activation of ERK signaling; thus, VPA ameliorated the anti-tumor effect of MgCl_2_. Although further studies are warranted, the combination treatment of MgCl_2_ with VPA is an effective strategy to improve the outcome of chemotherapy.

## Data Availability Statement

The original contributions presented in the study are included in the article/**Supplementary Material**. Further inquiries can be directed to the corresponding authors.

## Ethics Statement

The animal study was reviewed and approved by Animal Care and Use Committee of the Northeastern University Committee, China.

## Author Contributions

YH, BW, YR, and GQ conceived and designed the experiments. YH, TL, YY, HS, YC, XL, and ZH performed the experiments and analyzed the data. YH wrote the manuscript. TL and BW reviewed the manuscript. All authors contributed to the article and approved the submitted version.

## Funding

This work was funded by the National Natural Science Foundation of China (Nos.81502582, U1603125, and 31670770). Fund was also provided by the Fundamental Research Funds for the Central Universities (N182004002 and N141008001-8), the Program for JLU Science and Technology Innovative Research Team (2017TD-28), Liaoning Revitalization Talents Program (XLYC1902063) and Key Research and Development Plan of Liaoning Province (2020JH2/10300080).

## Conflict of Interest

The authors declare that the research was conducted in the absence of any commercial or financial relationships that could be construed as a potential conflict of interest.
