# Supplementary material for: Magnesium in Combinatorial With Valproic Acid Suppressed the Proliferation and Migration of Human Bladder Cancer Cells
Source: Front Oncol. 2020 Dec 11;10:589112. doi: 10.3389/fonc.2020.589112 (PMC7759627; doi:10.3389/fonc.2020.589112)

Figure S1. The survival rate of HEK-293 cells at different concentrations of MgCl2 for 24 h.

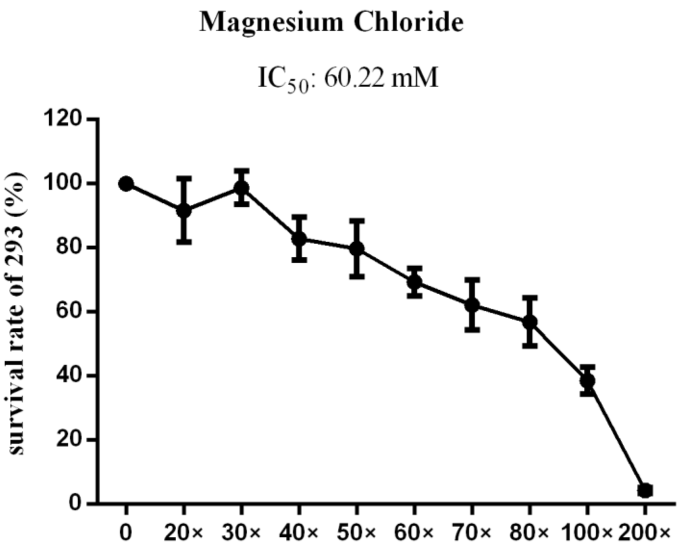

Figure S2. (A-D) Protein expression in $MgCl_2$ (42.5 mM) treated UC5 cells as revealed by Western blot analysis. (E) acH4K5 abundance in $MgCl_2$ (42.5 mM) treated UC3 cells as revealed by Western blot analysis.

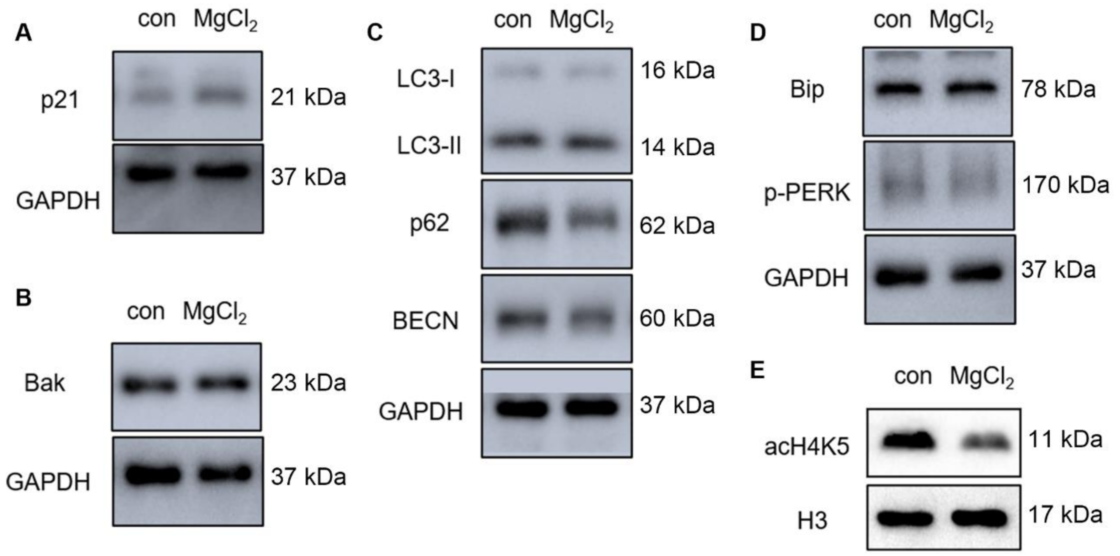

Figure S3. U0126 attenuated $MgCl_2$ (42.5 mM) induced apoptosis in UC3 bladder cancer cells. Cytosolic and mitochondrial protein fractions were collected using a Cell Mitochondria Isolation Kit (Beyotime, Shanghai, China). Western blot analysis was applied to detect apoptosis-related gene expression in cytosolic (A) and mitochondria (B) protein.

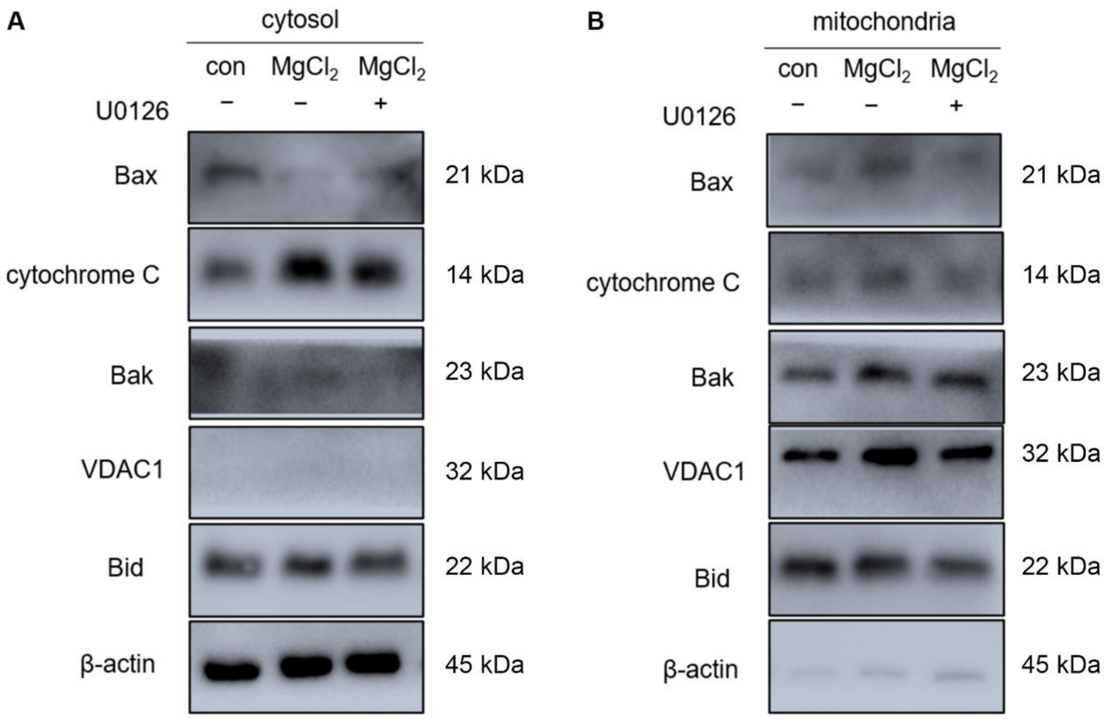

Figure S4. (A) The survival rate of UC5 cells with combination treatment as determined by CCK-8 assay. (B-D) Protein expression in UC5 cells with combinatorial treatment $MgCl_2$ (42.5 mM) and VPA (5 mM) as revealed by Western blot analysis.

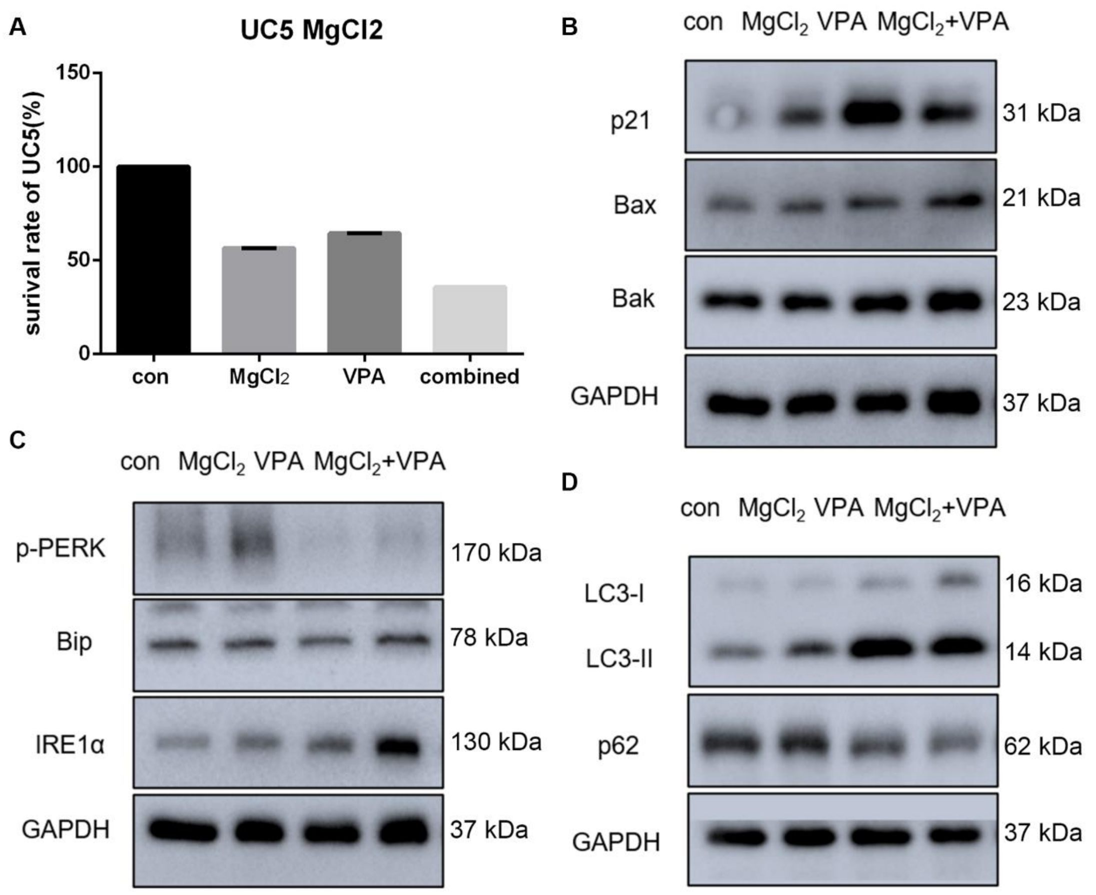

Figure S5. mRNA expression UC3 cells with combinatorial treatment as determined by qRT-PCR. 42.5 mM $MgCl_2$, 5 mM VPA and 0.05 mM STF083010 were applied for cell treatment. The differences among multiple groups were assessed using the Analysis of variance (ANOVA). *p<0.05 versus control, and **p<0.01 versus control.

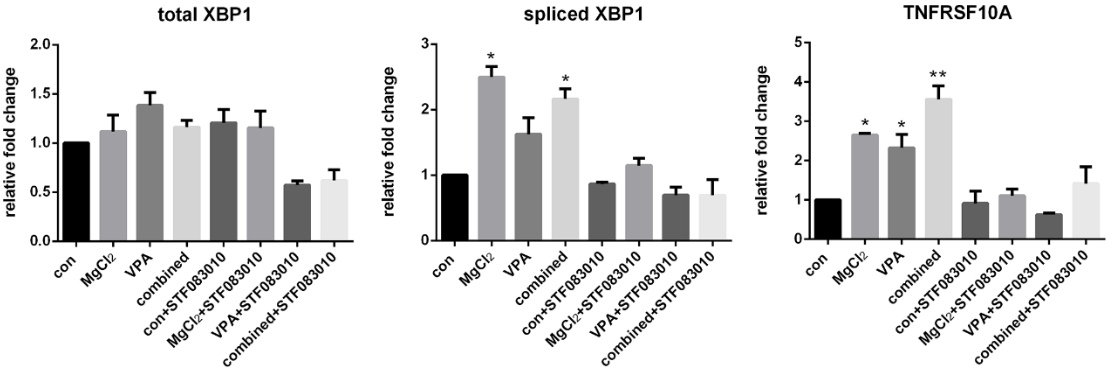

Supplement: Supplementary file 1 [file DataSheet_1.pdf]
