# Supplementary material for: Magnesium in Combinatorial With Valproic Acid Suppressed the Proliferation and Migration of Human Bladder Cancer Cells
Source: Front Oncol. 2020 Dec 11;10:589112. doi: 10.3389/fonc.2020.589112 (PMC7759627; doi:10.3389/fonc.2020.589112)
Supplement: Supplementary file 2 [file Table_1.docx]

**Supplementary Table S1. Primer sequences for qRT-PCR**

| **Genes** | **Forward** | **Reverse** |
| --- | --- | --- |
| p16 | TAGTATTGTATTAGGTAGGGGCGC | TATCGATAACCCGAAAAACGTT |
| p21 | GAGGCCGGGATGAGTTGGGAGGAG | CAGCCGGCGTTTGGAGTGGTAGAA |
| HRK | CCTACTGGCCTTGGCTGTG | TACAAGTTCCGCCTGCCG |
| FAS | GACCCAGAATACCAAGTGCAG | GTTCTGCTGTGTCTTGGACATTGTC |
| TNFRSF10A | ACACCCAGCAAAGTGTGG | CCGACGACGACAAACTTG |
| TNFRSF10B | GACTATAGCACTCACTGGAATGACC | GTCATCGAAGCACTGTCTCAGAG |
| ARTC1 | GGCCTCATGGAAGCACTTCA | GAGAAGAGGTCTCGTCGTGTGA |
| GRP78/Bip | ACGTGGAATGACCCGTCTGT | AACCACCTTGAACGGCAAGA |
| IRE1α | CGGGAGAACATCACTGTCCC | CCCGGTAGTGGTGCTTCTTA |
| TRAF2 | AAAGGGTCAGGAAGCCGTAG | AAAGGGTCAGGAAGCCGTAG |
| eIF | GACCTCGAGATGCCGGGGCTAAGTTG | GACAAGCTTATCTTCTTTATCTTCAGC |
| ERdj4 | TCTTAGGTGTGCCAAAATCGG | TGTCAGGGTGGTACTTCATGG |
| EDEM1 | CGGACGAGTACGAGAAGCG | CGTAGCCAAAGACGAACATGC |
| P58IPK | GGCTCGGTATTCCCCTTCCT | AGTAGCCCTCCGATAATAAGCAA |
| Total XBP1 | TTGTCACCCCTCCAGAACATC | TCCAGAATGCCCAACAGGAT |
| Spliced XBP1 | TGCTGAGTCCGCAGCAGGTG | GCTGGCAGGCTCTGGGGAAG |
| ATG3 | GCCGTTAAAGAGATCACACTGG | CATAGCCAAACAACCATAATCGTGG |
| ATG5 | CAGCTCTTCCTTGGAACATC | GGCTGTGGGATGATACTAATATG |
| BECN | GAAGACGTGGAAAAGAACCGC | CAGCCTGAAGTTATTGATTGTGC |
| RIPK1 | GCACCGCTAAGAAGAATGG | GCCACACAATCAAGTTGAAGAG |
| PARP-1 | CTCAGGGGAGGGTCTGATGA | CTTTGACACTGTGCTTGCCC |
| MLKL | GCTGAGTGATGTCTGGAAGG | CTTTGGAATCGTCCTCTGGG |
| SFRP2 | ATGATGATGACAACGACATAATG | GAGCCACAGCACCGATTT |
| WIF-1 | AATGCCAATGTCAAGAAGG | GATGTCGGAGTTCACCAGA |
| Wnt3a | TGCATAGGCTCCTTCCTGTGG | TGGCTGGTGGGCTGAATTTC |
| Wnt5a | GAGTGCTCGCATCCTCAT | GCATGTCTTCAGGCTACA |
| APC | CCAACAAGGCTACGCTAT | CTGCTCGCCAAGACAAAT |
| LEF1 | CAGTCATCCCGAAGAGGAAG | GCTCCTGAGAGGTTTGTGCT |
| c-Myc | TCAGAGGTGCCACGTCTCC | TCTTGGCAGCAGGATAGTCCTT |
| H-Ras | GCAATTTATGCTGCCGAATCTC | CAGCTATGGCATCCCCTACATT |
| N-Ras | AGCAGTGACGATGGCACTCAA | GGCATCAGTGCAGCTTCAAAGT |
| Raf-1 | GAAAGCCATCCACACAGGACA | TGCCAGTTTTGCACATGGAG |
| MEK2 | TTGATGAAGGCGTGGTTCATC | CCAGTGGTGTGTTCAGCTCAGA |
| Erk1 | GCGTTACATGTGGCAGCTTGA | TGGAACCCCACCCCATTTT |
| ABCG2 | CCTGAGATCCTGAGCCTTTG | CATTGGTGTTTCCTTG |
| CD44 | GGGATTGGTTTTCATGGTTG | GTGTGGTTGAAATGGTGCTG |
| CD133 | ATGACAAGCCCATCACAACA | AGCACTACCCAGAGACCAATG |
| CDK1 | GTCCGCAACAGGGAAGAACAG | CGAAAGCCAAGATAAGCAACTCC |
| cyclinB2 | ACAAGTCCACTCCAAGTTTAGGC | CCAAGAGCAGAGCAGTAATCCC |
| cyclinB1 | CTGTTGGTTTCTGCTGGGTGTAG | CGCCTGCCATGTTGATCTTCG |
| Caspase9 | TGTCCTACTCTACTTTCCCAGGTTTT | GTGAGCCCACTGCTCAAAGAT |
| CHOP | CAGAGCTGGAACCTGAGGAG | TGGATCAGTCTGGAAAAGCA |
| BAP31 | TTGCTGCTGTCCTTCCTGCTTAGA | ATGTACTTCTTGGCCGCCTCACTA |
| LC3 | GATGTCCGACTTATTCGAGAGC | TTGAGCTGTAAGCGCCTTCTA |
| VDAC1 | TGACGCCTGCTTCTCG | GCCACCAAGTTCTCCC |
| p62 | AGCTGCCCTCAGCCCTCTA | GGCTTCTCTTCCCTCCATGTT |
| GAPDH | CTTCGCTCTCTGCTCCTCC | CAATACGACCAAATCCGTTG |
