# Supplementary material for: Magnesium in Combinatorial With Valproic Acid Suppressed the Proliferation and Migration of Human Bladder Cancer Cells
Source: Front Oncol. 2020 Dec 11;10:589112. doi: 10.3389/fonc.2020.589112 (PMC7759627; doi:10.3389/fonc.2020.589112)
Supplement: Supplementary file 3 [file Table_2.docx]

Table S2. List of primary antibodies applied in this study

| Antibody | brand | dilution | application |
| --- | --- | --- | --- |
| p21 | Bimake | 1:1000 | Western blotting |
| cyclinB1 | Bimake | 1:1000 | Western blotting |
| Bak | Bimake | 1:1000 | Western blotting |
| MLKL | Bimake | 1:1000 | Western blotting |
| CDK1 | proteintech | 1:1000 | Western blotting |
| α-SMA | proteintech | 1:1000 | Western blotting |
| ZO-1 | proteintech | 1:1000 | Western blotting |
| MMP2 | proteintech | 1:1000 | Western blotting |
| MMP9 | proteintech | 1:1000 | Western blotting |
| CHOP | proteintech | 1:1000 | Western blotting |
| Bip | proteintech | 1:2000 | Western blotting |
| BECN | proteintech | 1:2000 | Western blotting |
| p62 | proteintech | 1:2000 | Western blotting |
| LC3 | proteintech | 1:1000 | Western blotting |
| Bax | Invitrogen | 1:1000 | Western blotting |
| Zeb1 | CST | 1:1000 | Western blotting |
| Vimentin | CST | 1:1000 | Western blotting |
| E-cadherin | CST | 1:1000 | Western blotting |
| Slug | CST | 1:1000 | Western blotting |
| VDAC | CST | 1:1000 | Western blotting |
| p-AKT | CST | 1:1000 | Western blotting |
| p-mTOR | CST | 1:1000 | Western blotting |
| mTOR | CST | 1:1000 | Western blotting |
| p-ERK | CST | 1:2000 | Western blotting |
| ERK | CST | 1:2000 | Western blotting |
| p-p38 | CST | 1:1000 | Western blotting |
| p38 | CST | 1:1000 | Western blotting |
| β-catenin | CST | 1:1000 | Western blotting |
| β-actin | CST | 1:2000 | Western blotting |
| GAPDH | CST | 1:5000 | Western blotting |
| FITC-conjugated antibody against CD44 | BD | 1:11 | FACS |
| PE-conjugated antibody against CD133 | BD | 1:11 | FACS |
